# Intraovarian regulation of gonadotropin-dependent folliculogenesis depends on notch receptor signaling pathways not involving Delta-like ligand 4 (Dll4)

**DOI:** 10.1186/1477-7827-11-43

**Published:** 2013-05-15

**Authors:** Vuk P Jovanovic, Christopher M Sauer, Carrie J Shawber, Raul Gomez, Xing Wang, Mark V Sauer, Jan Kitajewski, Ralf C Zimmermann

**Affiliations:** 1 Department of Obstetrics and Gynecology, Columbia University Medical Center, 622 W 168th Street, New York, NY 10032, USA; 2 Fertility Center of Langenhagen and Wolfsburg, Langenhagen 30861, Germany; 3 Yale University School of Medicine, New Haven, Connecticut, USA; 4 Fundacion IVI-Instituto Universitario IVI, INCLIVA, Universidad de Valencia, C/Guadassuar 1 Bajo, Valencia, 46015, Spain

**Keywords:** Notch, Dll4, Jagged, Folliculogenesis, Ovary, Gamma-secretase inhibitor, YW152F

## Abstract

**Background:**

In-situ hybridisation studies demonstrate that Notch receptors and ligands are expressed in granulosa cells (GCs) and in the theca layer vasculature of growing follicles. Notch signaling involves cell-to-cell interaction mediated by transmembrane receptors and ligands. This signaling pathway may represent a novel intraovarian regulator of gonadotropin-dependent follicular development to the preovulatory stage. We hypothesized that blocking Notch pathways would disrupt follicular maturation in the mouse ovary.

**Methods:**

Hypophysectomized CD21 female mice were administered pregnant mare serum gonadotropin (PMSG) for 3 days to stimulate follicular development. In one experiment, a pan-notch inhibitor, compound E, was initiated 2 days prior to and throughout stimulation (n = 10), while in a second experiment, a humanized phage Dll4 blocking antibody, *YW152F*, was used (n = 5). After sacrifice, ovarian histology, serum estradiol levels and uterine weights were compared to controls. The ovarian morphology was evaluated with hematoxylin/eosin staining and immunohistochemistry was performed for Notch1, Notch2, Notch3, Notch4, Jagged1, Dll4, platelet endothelial cell adhesion molecule (PECAM) and alpha-smooth muscle actin (α-SMA) detection.

**Results:**

We localized specific Notch ligands and receptors in the following structures: Dll4 is specific to theca layer endothelial cells (ECs); Notch1/Notch4 and Jagged1 are expressed in theca layer ECs and vascular smooth muscle cells (VSMCs), whereas Notch3 is restricted to VSMCs; Notch2 is expressed mostly on GCs of small follicles. Administration of a pan-Notch inhibitor, compound E, inhibits follicular development to the preovulatory stage (8.5 preovulatory follicles in treatment vs. 3.4 preovulatory follicles in control, p < 0.01; average number per ovary) with significant secondary effects on ovarian and uterine weight and estradiol secretion in a setting of uninhibited vascular proliferation, but disorganized appearance of ECs and VSMCs. Inhibition of endothelial Notch1 function through the inactivation of its ligand Dll4 with the blocking antibody YW152F induces mild disorganisation of follicular vasculature, but has no significant effect on gonadotropin-dependent folliculogenesis.

**Conclusions:**

Our experiments suggest that the complete blockage of the Notch signaling pathway with compound E impairs folliculogenesis and induces disruption of gonadotropin stimulated angiogenesis. It seems the mechanism involves Notch1 and Notch3, specifically, causing the improper assembly of ECs and VSMCs in the theca layer, although the potential role of non-angiogenic Notch signaling, such as Jagged2 to Notch2 in GCs, remains to be elucidated.

## Background

Major advances have been made in identifying and characterizing the role of intraovarian regulators such as insulin growth factor (IGF), epidermal growth factor (EGF), vascular endothelial growth factor (VEGF), transforming growth factors, anti-Müllerian hormone, bone morphogenetic protein with respect to gonadotropin-dependent follicular development [1]. Despite these advances, our understanding of how folliculogenesis is regulated is far from complete, which suggests the existence of other unidentified intraovarian regulators. In-situ hybridisation studies have shown that vascular and non-vascular components of the Notch pathway are localized to specific structures in the ovary [2,3]. For example m-RNA of Notch2, Notch3, and Jagged2 as well as downstream targets of Notch are highly expressed in the granulosa cells (GCs) of developing follicles [2]. Vascular Notch m-RNA (Notch1 and Notch4) was detected on blood vessels in the theca layer of growing follicles [2], a finding later validated by immunofluorescent studies [4]. Notch1 and the Notch ligand Jagged1 can be detected on ECs as well as vascular smooth muscle cells (VSMCs) [4]. The Notch ligands Dll1 and Dll3 are absent in the ovary [2], whereas the Notch1 ligand Dll4 was detected by in-situ hybridisation in ovarian vasculature [2,5]. Results derived from expression analysis suggest that Notch is a novel intraovarian regulator, which regulates folliculogenesis through vascular and non-vascular mechanisms [2,6]. It should be noted that Notch would be unique among intraovarian regulators as Notch ligands and receptors are single-pass transmembrane proteins, requiring a juxtacrine (or contact-dependent) signaling mechanism [3,7,8].

We hypothesized that blocking Notch pathways would disrupt *in-vivo* folliculogenesis in our mouse model by affecting vascular and non-vascular pathways. This would confirm the effects on folliculogenesis described *in vitro*[3], but also evaluate vascular growth disruption surrounding maturing follicles. We used a mouse model to perform functional studies using a pan-Notch inhibitor, compound E, as well as a blocking antibody (BAb) against the Notch1 ligand Dll4, located exclusively on endothelial cells (ECs). As in-situ hybridisation studies can be discrepant with localisation of the corresponding protein, we performed immunofluorescence with antibodies to Notch2, Notch3, and Dll4.

## Methods

The study was reviewed and approved by the Institutional Review Board and the Institutional Animal Care Committee of the Columbia University Medical Center.

### Animal model

CD21 female mice (Charles River, Wilmington, MA, USA), hypophysectomized before 22 days of life, were used for all experiments. Insignificant weight gain (< 2 g over 8 days after arrival) and low estrogenic state vaginal smears verified that the surgery had been successful in arresting follicular growth at the advanced preantral stage due to the absence of pituitary gonadotropin secretions [9].

### Experimental design

Experiment 1: Follicle development was stimulated in all mice with 20 IU of PMSG (ProSpec-Tany TechnoGene, Rehovot, Israel) for 3 days. Treatment group animals (n = 10) were injected intraperitoneally (i.p.) with the pan-notch inhibitor compound E (KRICT - Korean Research Institute of Chemical Technology, Daejeon, Korea) at a dose of 30 μmol/kg animal. Compound E was suspended finely in 0.5% (w/v) hydroxypropylmethylcellulose (Methocel E4M) and 0.1% (w/v) Tween 80 (both Sigma-Aldrich, St. Louis, MO, USA) in water [10]. The compound E was freshly prepared and injected for 5 days starting 2 days prior to the PMSG injection. All treatment animals were administered Dimethyl sulfoxide (DMSO) (Sigma-Aldrich, St. Louis, MO, USA) with the compound E suspension mixed to a total i.p. injection volume of 170 μL. Control group animals (n = 10) were injected i.p. with 170 μL DMSO alone. One hour before sacrifice all animals were injected i.p. with 1 ml 5-bromo-2-deoxyuridine (BrdU) reagent (Zymed-Invitrogen, Camarillo, CA USA) per 100 g mouse [9].

Experiment 2: Treatment group animals (n = 5) were injected with the Genentech anti-Dll4 blocking antibody (BAb) YW152F (10 mg kg^-1^) [11] 1 day prior and 1 day after PMSG administration. The antibodies were diluted in a total volume of 170 μL DMSO and the solution was administered i.p. Control animals (n = 5) were injected with human IgG using the same dose and regimen. Performance of the experiment was otherwise done as described in experiment 1.

### Histology

All animals were sacrificed 5 days after the initiation of compound E or DMSO treatment and 4 days after anti-Dll4 BAb YW152F administration. Both ovaries and the uterus were removed and weighed. Ovaries were embedded in optimal cutting temperature (OCT) medium (Thermo Fisher Scientific, Waltham, MA, USA), flash frozen and stored at -80°C. One whole ovary was sectioned serially (12 μm), and each section was stained with hematoxylin/eosin (H&E) to count the total number of gonadotropin-dependent preovulatory follicles per ovary as described previously [9]. Sections of the contra-lateral ovary (7 μm) of each mouse were used for specific immunohistochemistry [9]. A piece of small intestine was flushed gently with cold phosphate buffered saline (PBS) followed by a flush of formalin. The tissue was then fixed in formalin at 21°C for 16 h. Intestinal sections were stained with periodic acid-Shiff (PAS) staining (Sigma-Aldrich, St. Louis, MO, USA) in order to detect goblet cells, since Notch γ-secretase inhibition turns proliferative cells in intestinal crypts into goblet cells [10]. An increase in the number of goblet cells in the treatment group over control group served as a positive control demonstrating that compound E is active. Intestines from animals of experiment 2 were not stained for goblet cells as they are not affected by anti-Dll4 antibodies [11]. Blood was obtained through cardiopuncture for the measurement of estradiol (E2) levels as described previously [9].

### Immunohistochemistry

The primary antibodies used in these assays were as follows: goat anti-Notch1 antibody diluted 1/1000, goat anti-Notch2 diluted 1/500, goat anti-Notch3 antibody diluted 1/1000, rat anti-Notch4 antibody diluted 1/500, goat anti-Jagged1 antibody diluted 1/500, goat anti-Dll4 diluted 1/200 (all R&D, Minneapolis, MN, USA), monoclonal rat anti-PECAM diluted 1/200 (Pharmingen BD Biosciences, San Jose, CA, USA), and a mouse anti-alpha smooth muscle actin antibody diluted 1/200 (VSMC marker, Sigma-Aldrich, St. Louis, MO, USA). The secondary anti-goat, anti-rat, anti-mouse 488-alexa and 594-alexa were applied at dilution 1/1000 and finally mounted with DAPI antibodies (all Sigma-Aldrich, St. Louis, MO, USA). Immunofluorescence and BrdU staining (Zymed-Invitrogen, Camarillo, CA USA) was performed using standard immunohistochemistry and immunofluorescence protocols. [4,9].

### Data analysis

For each animal, all H&E sections from one ovary were evaluated to count the total number of preovulatory follicles per ovary as previously described [9]. Statistical analysis was performed using the Statistical Package for Social Science version 15 (SPSS, Inc., Chicago, IL, USA). Data are expressed as mean ± standard error (SE). We used an unpaired t-test to compare sample means with statistical significance defined as p < 0.05.

## Results

### Immunofluorescent studies

Notch2 is expressed in GCs of small follicles; Notch3 and Dll4 are expressed in follicular vasculature.

**Figure 1:**
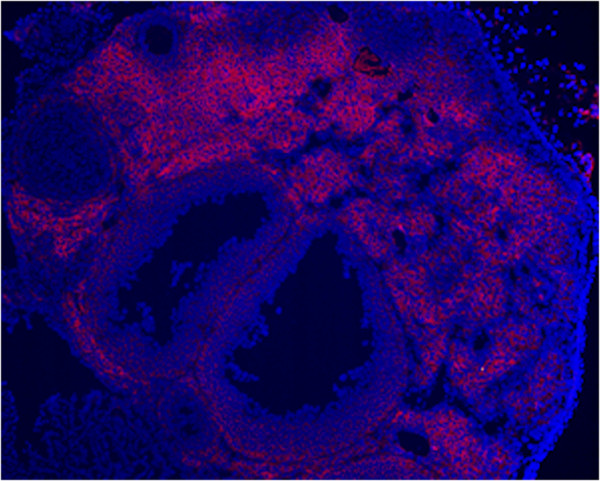
**Notch2 (red) in developing follicles. **Intense staining in secondary follicles and sporadic red staining in tertiary follicles with complete absence of staining in the peripheral theca layer of the depicted 2 large follicles (10× magnification, DAPI counterstaining in blue).

**Figure 2:**
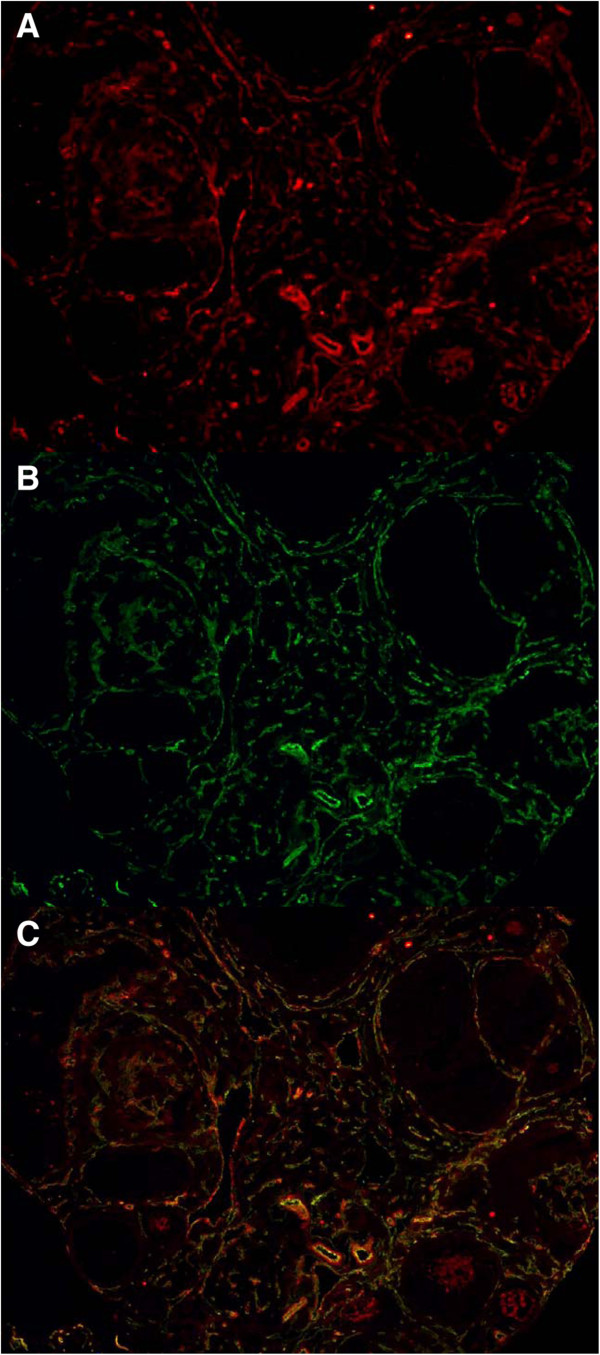
**PMSG stimulated untreated ovary with tertiary follicles: A) Notch3 in red B) PECAM in green C) Composite image with overlapping areas in yellow. **Please note presence of Notch3 staining in the peripheral theca layer and absence of Notch3 staining on granulosa cells. (10× magnification).

**Figure 3:**
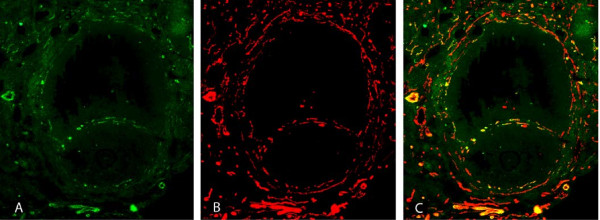
Doublestaining of a developing tertiary follicle: A) Dll4 shown in green B) PECAM in red C) composite overlapping images showing presence of Dll4 in endothelial cells of the theca vasculature layer.

Using immunofluorescent analysis, we found that Notch2 is expressed in GCs of secondary follicles and sporadically in GCs of preovulatory follicles, but is absent in the peripheral theca layer (Figure 1). Notch3 expression is largely restricted to VSMCs located in the theca layer of growing follicles and in interstitial tissue (Figure 2). No evidence of Notch3 expression was seen in follicular GCs. The Notch1/Notch4 ligand, Dll4, is expressed exclusively in ECs in the follicular theca layer vasculature and ovarian interstitial tissue during the follicular phase (Figure 3). Previously reported vascular expression patterns of Notch1 (ECs and VSMCs), Notch4 (ECs and VSMCs), and Jagged1 (ECs and VSMCs) [4] were confirmed (data not shown).

### Functional studies

#### Compound E

The pan-Notch inhibitor, compound E, inhibits gonadotropin-dependent follicle development to the preovulatory stage.

**Figure 4:**
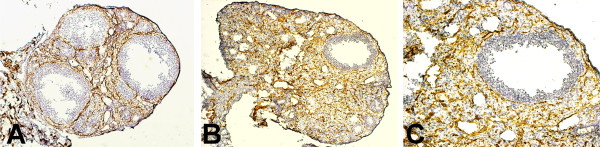
**Inhibition of Notch signaling with a γ-secretase inhibitor (Compound E) blocked follicle development to the preovulatory stage. **Immunohistochemistry for PECAM (brown) counterstained with hematoxylin. Please note disorganized appearance of PECAM staining in treatment group. **A**) three control preovulatory follicles (10× magnification) **B**) γ-secretase inhibitor treated follicles (10× magnification) **C**) Enlargement of treatment follicle in **B** (20 × magnification).

Administration of the pan-Notch inhibitor, compound E [12], induced a decrease in the number of follicles maturing to the preovulatory stage when compared to control after gonadotropin stimulation: control group: 8.5 ± 0.7 (mean ± SEM); treatment group: 3.8 ± 0.8 (p < 0.01) (Figure 4, Table 1). In addition, the growing follicles in the treatment group were smaller in size and irregular in shape (Figure 4). The mean plasma E2 level in the control group was 83.4 ± 6.5 pg/mL, whereas in the treatment group it was 29.3 ± 5.2 pg/mL (p < 0.01). Consistent with a lower number of follicles in the ovaries in the treatment group, the mean ovarian weight was significantly lower in the animals treated with compound E (control 5.8 ± 0.8 mg versus treatment 3.5 ± 0.3 mg; p < 0.01). Uterine weight, reflecting estrogen activity, was also lower in the treatment group (control 42.9 ± 6.7 mg versus treatment 32.3 ± 2.3 mg; p < 0.05), as shown in Table 1.

**Table 1:** Effect of compound E administration on total pre-ovulatory follicle number, mean ovarian and uterine weights and serum estradiol levels when compared to control animals

	**Control (n = 10)**	**Treatment (n = 10)**	**P-value**
*Follicle count*	8.5 ± 0.7	3.8 ± 0.8	<0.01
*Ovary (mg)*	5.8 ± 0.8	3.5 ± 0.3	<0.01
*Uterus (mg)*	42.9 ± 6.7	32.3 ± 2.3	<0.05
*Estradiol (pg/mL)*	83.4 ± 6.5	29 ± 5.2	<0.01

Blocking Notch signaling with compound E results in follicular and interstitial tissue blood vessel disorganization and does not block cell proliferation.

**Figure 5:**
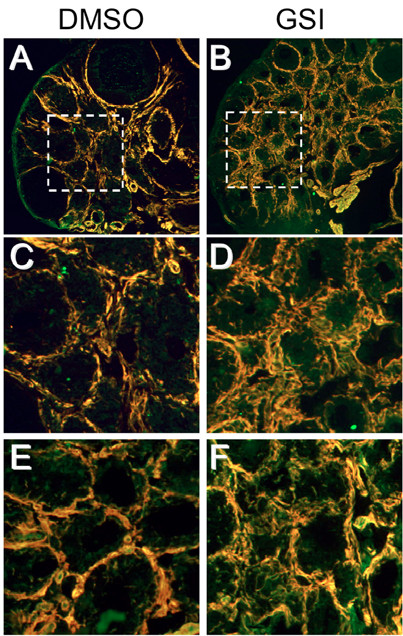
**Compound E inhibition of Notch signaling increased vascular smooth muscle cells (VSMC) density and altered VSMC organization in the ovarian stroma. A**-**D**) Immunofluorescence staining (10× magnified) for Notch3 (green) and α-SMA (red). **C**, **D**) enlargement of boxes areas in upper panels (20× magnified). Please note increased thickness and disorganized appearance of VSMCs in **B **and **D**. **E**, **F**) PECAM (green) and α-SMA (red). Please note disorganisation and failure to overlap in F (20× magnified).

**Figure 6:**
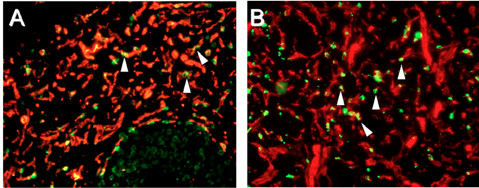
**Cell proliferation. **PECAM (red) and BrdU (green). **A**) DMSO control treated **B**) Compound E, (20 × magnification). Please note that proliferation was not blocked by compound E (**B**) and even might be increased over control.

The density of VSMCs expressing alpha-smooth muscle actin (α-SMA) in the theca layer of follicles and interstitial tissue of compound E treated animals (Figure 5A-D: Notch3 and α-SMA) was increased when compared to control. VSMCs had a very disorganized appearance with increased vascular thickness when compared to control (Figure 5A). VSMCs continuity surrounding individual follicles was often disrupted (Figure 5B). A similar pattern of disorganization was seen for endothelial cells with an increase in density in the treatment group when compared to control (Figure 5E and 5F). Double staining for PECAM and α-SMA showed mostly an organized pattern of overlap in the control group (Figure 5E) as described previously [4]. In contrast to the treatment group, many endothelial cells (green) are devoid of adjacent VSMCs (Figure 5F). Proliferation of non-GCs, representing mostly dividing endothelial cells and VSMCs, was detected (Figure 6) demonstrating that compound E did not stop angiogenic proliferation. When comparing proliferation to the control group, it appears that vascular proliferation might even be increased in the treatment group (Figure 6), possibly explaining the increase in vascular density seen in compound E treated ovaries. Therefore, inhibition of gonadotropin-dependent follicle growth occurs in a setting of ongoing angiogenesis.

Goblet cells in the intestine are increased in compound E treated animals.

**Figure 7:**
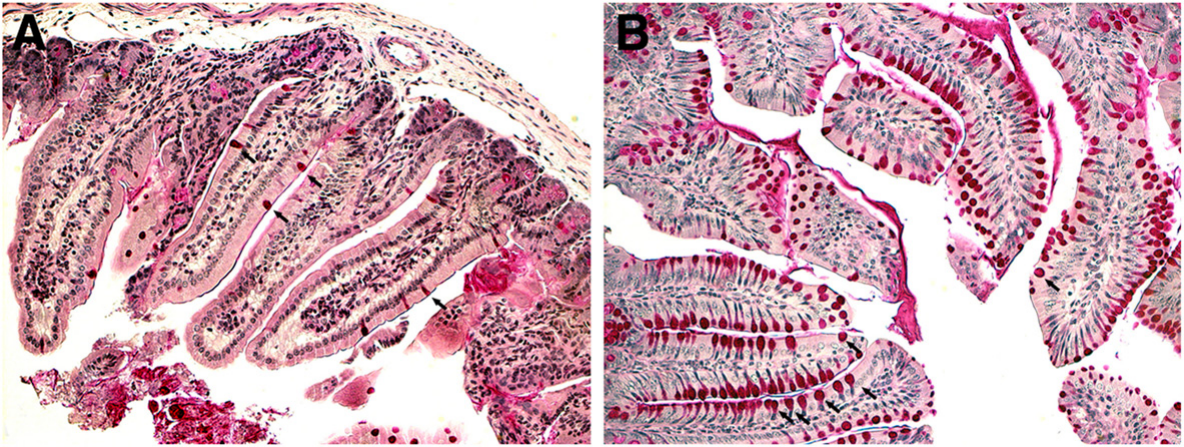
**Intestinal section: A) control (DMSO), B) treatment (compound E). **Goblet cells in red. Please note the increase of intestinal goblet cells in **B** over **A** (20× magnification).

There was an increase in goblet cells in the intestines of all compound E treated animals (Figure 7), verifying that compound E was active.

#### Dll4 Blocking antibody YW152F

Dll4 Blocking Antibody YW152F does not inhibit gonadotropin-dependent follicle development to the preovulatory stage.

**Figure 8:**
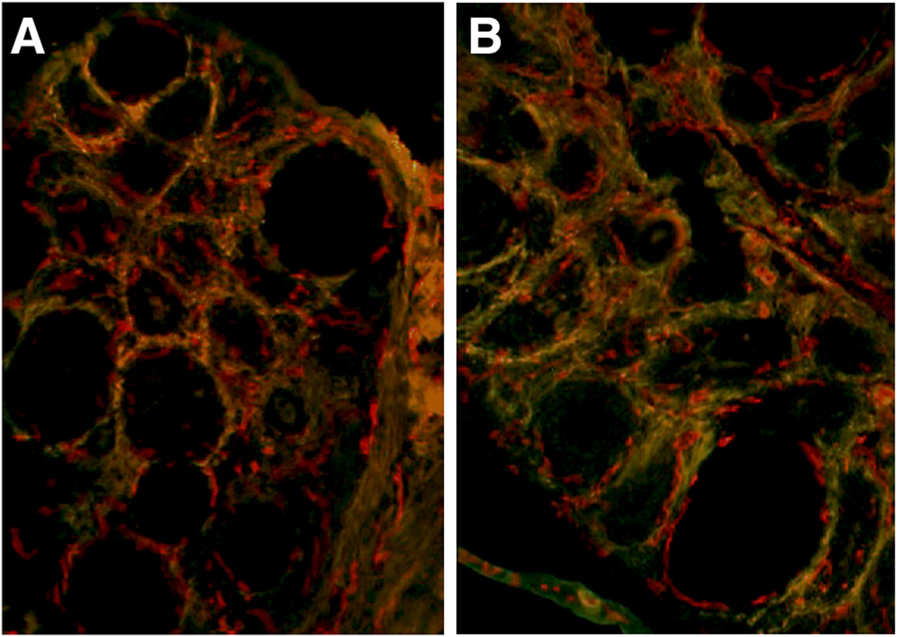
**Control and Dll4 blocking antibody YW152F treated ovaries stimulated with PMSG (10× magnification). **Doublestaining with PECAM (red) and a-SMA (green). **A**) Control ovary. **B**) YW152F treated ovary. Please note that vascular appearance is slightly more disorganized in **B**.

Administration of Dll4 BAb YW152F did not cause a decrease in the number of follicles maturing to the preovulatory stage when compared to control after gonadotropin stimulation: control group: 9.2 ± 0.5 (mean ± SEM); treatment group: 8.7 ± 0.7 (p > 0.05) (Figure 8). The mean plasma E2 level in the control group was 78.6 ± 5.4 pg/mL, whereas in the treatment group it was 69.4 ± 4.9 pg/mL (p > 0.05). Mean ovarian and uterine weights were not different between the 2 groups (ovary: control 6.2 ± 0.7 mg versus treatment 5.5 ± 0.4 mg, p > 0.05; uterus: control 40.1 ± 5.9 mg versus treatment 38.7 ± 4.4 mg, p > 0.05; Table 2). Analysis of follicular vasculature demonstrates that integrity is maintained during the treatment, even though it has a slightly disorganized appearance (Figure 8).

**Table 2:** Effect of Dll4 blocking antibody YW152F on total pre-ovulatory follicle number, mean ovarian and uterine weights and serum estradiol levels when compared to control animals

	**Control (n = 5)**	**Treatment (n = 5)**	**P-value**
*Follicle count*	9.2 ± 0.5	8.7 ± 0.7	n.s.
*Ovary (mg)*	6.2 ± 0.7	5.5 ± 0.3	n.s.
*Uterus (mg)*	40.1 ± 5.9	38.7 ± 4.4	n.s.
*Estradiol (pg/mL)*	78.6 ± 5.4	69.4 ± 4.9	n.s.

n.s. not significant.

## Discussion

To understand the possible treatment effects of interrupting Notch signaling with compound E or an anti-Dll4 BAb on gonadotropin-dependent folliculogenesis, one has to have a good understanding of where these molecules are expressed within the follicles. Complementary analysis of the expression of the Notch family proteins combined with preexisting data [2,4,5] has allowed us to obtain a better idea about which type of cell-to-cell Notch signalling occurs in growing follicles. We demonstrated that Notch3 is expressed exclusively in vascular smooth muscle cells (VSMCs), which are adjacent to theca layer endothelial cells [4]. The presence of Notch3 together with Notch1 [4] on VSMCs suggests a role in organizing the formation of a mature vasculature. It is very likely to involve interaction with the Notch ligand Jagged1 [7], which is expressed on ECs and VSMCs in the theca layer of growing follicles [4]. It remains unclear as to why we were unable to detect Notch3 in GCs as described by Johnson et al. [2]. Notch2 was consistently expressed in GCs of preantral and small antral follicles and sporadic Notch2 staining was also seen in preovulatory follicles. These findings suggest that Notch2 in GCs is activated by neighbouring GCs expressing Jagged2 [2], although we did not specifically stain for this protein. Our findings confirm the localization noted in *in vitro* models [3]. Dll4 is exclusively expressed on ECs. Based on previous results [4,5] and consistent with our data, this suggests that Dll4 expressed on ECs signals to a neighboring EC expressing Notch1 and possibly Notch4. As Jagged1 is present on ECs, it might not only signal to VSMCs Notch1/Notch3, but also compete with Dll4 regarding the interaction with the Notch1 receptor located on neighboring ECs, as suggested previously by Benedito [13].

Inhibition of Notch function with the γ-secretase inhibitor compound E significantly blocked gonadotropin-dependent follicle growth up to the preovulatory stage of development. Thus, the number of follicles evolving to the preovulatory stage was significantly decreased. Due to the blockage of gonadotropin-dependent follicle development, the following secondary effects were seen: 1) lesser degree of increase in ovarian weight due to the inability to develop tertiary follicles similar in number to control; 2) lesser degree of increase in uterine weight due to lower E2 secretion in the treatment group when compared to control. In contrast to the effects of VEGF receptor 2 (VEGFR-2) BAb on gonadotropin-dependent folliculogenesis [9], no reduction in follicular or interstitial area blood vessels is seen in ovaries subjected to compound E. Even though we did not quantify ECs or VSMCs, our visual inspection suggests that there might be a slight increase of these cell types in the treatment group. This supports the finding that vascular cell proliferation continued to occur at least at a level similar to control in the ovaries from compound E treated animals.

The salient feature of ovarian vasculature exposed to a γ-secretase inhibitor is its disorganized appearance. One has the impression that ECs and VSMCs have lost the ability to connect in an orderly fashion during angiogenesis. These observations may suggest that compound E induced perturbation of angiogenesis did not allow proper assembly of blood vessels.

It is of high interest that disruption of EC signaling through YW152F, an anti-Dll4 BAb [11] did not disrupt follicle growth to the preovulatory stage, nor did it affect ovarian or uterine weight or E2 production or secretion. The blocking of EC Notch1 activation seems to cause a mild level of disorganization of the interaction of ECs and VSMCs, but it is insufficient to block functional vascular growth and blood circulation to support follicle development to the preovulatory stage. In the retina, YW152F creates a phenotype of non-productive sprouting angiogenesis [11], which is very similar to the effects seen with γ-secretase inhbitors.

The weakness of our YW152F experiment is that one could argue that the absence of inhibiting effect on folliculogenesis in the treated animals might be due to ineffectiveness of the administered Dll4 BAb. Unlike with compound E, where the effect can be validated by observing goblet cell proliferation in the gut, there is no such readily available positive control for the YW152F treated animals. However, when administering YW152F during corpus luteum formation in the same animal model, there are profound differences in angiogenesis when Dll4 is blocked [14]. This can indirectly serve as a proof of action and suggests that different types of angiogenic development and growth occur in follicular and luteal phase, indicating that circular elongation angiogenesis observed during follicular growth is quite different from sprouting angiogenesis in other tissues.

As Notch function is complex, several possibilities exist to explain our results at the molecular level.

### Notch and angiogenesis

During inhibition of Notch function, through compound E or YW152F, PMSG driven VEGF production in GCs is maintained to stimulate vascular growth by activation of VEGFR-2 on endothelial cells [9,15,16]. Disruption of endothelial Notch1 signaling through blockage of Dll4 is not sufficient to disrupt coordination of vascular growth in a significant way. In contrast, interference with Notch1 signaling on endothelial cells, as well as Notch1 and Notch3 signaling on VSMCs in compound E treated animals disrupts critical coordination between these 2 cell types, which is necessary to form mature functional vasculature required for gonadotropin-dependent follicular growth. These observations suggest that Notch1 and Notch3 coordinate VEGF driven angiogenesis in the theca layer during gonadotropin-dependent folliculogenesis.

### Effects of notch on non-angiogenic cells in the ovary

In-situ hybridization studies demonstrate that Notch2 and Notch3 are expressed on GCs [2]. We did not detect Notch3 on GCs, but did see Notch2 expressed. Johnson et al. speculated that GCs Notch activity is necessary for proliferation and differentiation, while preventing follicular atresia due to apoptosis. *In vitro* models have shown that inhibition of Notch2 leads to reduction of c-Myc inhibiting granulosa proliferation. Therefore, we suggest that blockage of Notch2 through administration of compound E might have affected GCs proliferation and differentiation, which in turn could have contributed to the inhibition of follicle development. In this case, the absence of significant effects observed in YW152F treated animals would be plausible, since our immunohistochemistry stains did not demonstrate presence of Dll4 or Notch3 within the follicle and blocking this pathway may have no impact on notch signaling among granulosa cells. Thus, further experiments with specific inhibition of Notch2 and Jagged2 are needed.

## Conclusions

In summary, we demonstrated by immunohistochemistry that members of the Notch family are expressed primarily in the vasculature (except Notch2) of follicles during folliculogenesis to the preovulatory stage, and therefore represent a new group of intraovarian regulators. Among intraovarian regulators, Notch is unique as the ligand and receptor are single-pass transmembrane proteins, which restricts the Notch pathway to signaling to neighboring cells [7]. Through functional studies we demonstrated that compound E, a pan-Notch inhibitor, but not YW152F, a Dll4 blocking antibody, disrupts the assembly of theca layer ECs with VSMCs enough to diminish gonadotropin-dependent follicle growth. It is meaningful that this type of vascular disturbance is distinctly different from the non-productive sprouting angiogenesis seen in the retina when exposed to γ-secretase inhibitors. It is likely that non-angiogenic Notch2 detected on GCs also plays a role in gonadotropin-dependent folliculogenesis. Our results represent a preliminary attempt to determine that vascular and possibly non-vascular Notch play an important role during gonadotropin-dependent follicle growth to the preovulatory stage of development.

## Competing interests

The authors declare that they have no competing interests.

## Authors’ contributions

VPJ and CMS carried out all laboratory experiments, analyzed the data and interpreted the results. VPJ and RCZ drafted the manuscript edited by CMS and VMS. CJS assisted in data analysis and optimization of immunohistochemistry. RG performed BrdU staining and assisted in animal experiments. XW assisted in preparation and administration of Compound E solution. JK provided material support and participated in design and coordination. RCZ conceived and implemented the study design. All authors read and approved the final manuscript.
